# Silk fibroin hydrogel scaffolds incorporated with chitosan nanoparticles repair articular cartilage defects by regulating TGF-β1 and BMP-2

**DOI:** 10.1186/s13075-020-02382-x

**Published:** 2021-02-02

**Authors:** Yuan Li, Yanping Liu, Qiang Guo

**Affiliations:** 1 Department of Joint Surgery, Linyi People’s Hospital, Linyi, 276000 People’s Republic of China; 2 Department of Orthopaedics of Integrated traditional and Western Medicine, Linyi People’s Hospital, Linyi, 276000 People’s Republic of China; 3 Department of Hand and Foot Surgery, Linyi People’s Hospital, Linyi, 276000 People’s Republic of China

**Keywords:** Hydrogel, Transforming growth factor-β1, Bone morphogenetic protein-2, Tissue-engineered cartilage, Articular cartilage defects

## Abstract

Cartilage defects frequently occur around the knee joint yet cartilage has limited self-repair abilities. Hydrogel scaffolds have excellent potential for use in tissue engineering. Therefore, the aim of the present study was to assess the ability of silk fibroin (SF) hydrogel scaffolds incorporated with chitosan (CS) nanoparticles (NPs) to repair knee joint cartilage defects. In the present study, composite systems of CS NPs incorporated with transforming growth factor-β1 (TGF-β1; TGF-β1@CS) and SF incorporated with bone morphogenetic protein-2 (BMP-2; TGF-β1@CS/BMP-2@SF) were developed and characterized with respect to their size distribution, zeta potential, morphology, and release of TGF-β1 and BMP-2. Bone marrow stromal cells (BMSCs) were co-cultured with TGF-β1@CS/BMP-2@SF extracts to assess chondrogenesis in vitro using a cell counting kit-8 assay, which was followed by in vivo evaluations in a rabbit model of knee joint cartilage defects. The constructed TGF-β1@CS/BMP-2@SF composite system was successfully characterized and showed favorable biocompatibility. In the presence of TGF-β1@CS/BMP-2@SF extracts, BMSCs exhibited normal cell morphology and enhanced chondrogenic ability both in vitro and in vivo, as evidenced by the promotion of cell viability and the alleviation of cartilage defects. Thus, the TGF-β1@CS/BMP-2@SF hydrogel developed in the present study promoted chondrogenic ability of BMSCs both in vivo and in vitro by releasing TGF-β1 and BMP-2, thereby offering a novel therapeutic strategy for repairing articular cartilage defects in knee joints.

## Introduction

Hyaline articular cartilage is crucial to the normal function of the knee joint while focal cartilage defects can further develop into diffuse osteoarthritis if untreated [1]. Currently, tissue grafting has been considered as the principal therapeutic strategy to bone regeneration and large bone defect repair, yet there are still limitations, including disease transmission and donor site morbidity, suggesting the need of exploring combining biomaterial scaffolds with biochemical cues [2].

Tissue engineering, including scaffold construction, has been reviewed as a promising treatment modality for articular cartilage defects in order to restore joint motion, relieve pain, and delay the occurrence of osteoarthritis [3]. During the application of tissue engineering, silk fibroin (SF), a fibrous protein mostly generated by silkworms and spiders, has been demonstrated as a desirable scaffold material with regard to its property to support the differentiation of mesenchymal stromal/stem cells (MSCs) [4]. SF microparticles have been identified to be functional for sustained delivery of various bone morphogenetic proteins (BMPs) and cytokines beneficial to new bone formation [5]. Importantly, a delivery system based on silk hydrogel has been reported to carry BMPs to manage large bone defects [2]. Furthermore, SF has been indicated to form composites by combining with a variety of nanoparticles (NPs) characterized by superior biomedical application performance [6]. Chitosan (CS) NPs have been recognized with great physiochemical properties to function as vehicles of drugs [7]. In addition, modular CS hydrogel incorporated with transforming growth factor-β1 (TGF-β1), a key player in chondrogenesis promotion, has been documented to induce therapeutic effects on articular cartilage defects [8]. Also, the interplay between bone morphogenetic protein-2 (BMP-2) and TGF-β1 in different types of CS hydrogels has been suggested to have clinical potentials to promote bone formation and regeneration of cartilage-bone interface [9, 10].

Notably, SF/CS thin composite film has been used for bone injury repair and tissue engineering scaffold of bone and cartilage, providing a comparable environment for osteogenic and adipogenic differentiation of bone mesenchymal stromal/stem cells (BMSCs) in rats [11]. BMSCs have emerged as a novel option for repair of articular cartilage defects featured with superior repair effect [12, 13]. For repair purpose, MSCs are required to differentiate into chondrocytes by attracting and responding to correct biological signals for the correct extracellular matrix generation, including a variety of growth factors [14]. Given the aforementioned evidence, we endeavored to probe into the protective effects conferred by combination of SF hydrogel and CS NPs on articular cartilage defects of the knee joints involving the delivery of TGF-β1 and BMP-2 both in vitro and in vivo.

## Materials and methods

### Ethics statement

The study was approved by the Ethics Committee of Linyi People’s Hospital and performed according to the Guide for the Care and Use of Laboratory Animals published by the US National Institutes of Health. Extensive efforts were made to ensure minimal suffering of the included animals.

### Preparation and characterization of TGF-β1-incorporated CS (TGF-β1@CS) NPs

Preparation of empty CS NPs: an appropriate amount of CS powder (the maximum decomposition temperature of CS was about 300 °C, and the molecular weight was 50kD) was weighed, dissolved in 1% acetic acid solution, and stirred to prepare 1 mg/mL CS solution, with the pH adjusted to about 5 using 2 mol/L NaOH. Sodium tripolyphosphate at different weights was weighed, dissolved in the same volume of ultrapure water, and dropped into the CS solution at a rate of 20 drops/min, followed by stirring and crosslinking for 30 min. Then, opalescent solution was obtained and subjected to centrifugation at 12500 rpm and 4 °C for 15 min to remove free TGF-β1. After reaction, the sample was filtered through a 0.45-μm filter membrane and cryopreserved.

### Preparation of CS NPs loaded with TGF-β1

CS solution was first prepared according to the above-mentioned method. Next, TGF-β1 solution was dissolved in the above CS solution with the weight ratio of 1:3. The following procedure was the same as above. The free-floating TGF-β1 was removed through centrifugation at 12500 rpm and 4 °C for 15 min.

The size distribution, zeta potential, and polymer dispersion index (PDI) of TGF-β1@CS NPs were examined by dynamic light scattering (DLS). The morphology was observed under a scanning electron microscope (SEM). Enzyme linked immunosorbent assay (ELISA) was applied to detect the release of TGF-β1 in vitro.

### Preparation and characterization of BMP-2-incorporated SF (BMP-2@SF) hydrogel

*Bombyx mori* (*B. mori*) silk was boiled in 0.02 M Na_2_CO_3_ solution for 30 min and dried overnight. The dried SF was dissolved in lithium bromide aqueous solution for 4 h and sealed in a dialysis bag (MW 3500), followed by dialysis in deionized water for 3 days to obtain SF solution.

BMP-2 was dissolved in SF to prepare BMP-2 SF solution at a final concentration of 2% (w/v). BMP-2@SF hydrogel was prepared using ultrasound-induced crosslinking, followed by morphology observation under the SEM and porosity calculation. The release of BMP-2 in vitro was detected using ELISA.

### Preparation and characterization of SF-incorporated CS-NPs composite hydrogel scaffolds (TGF-β1@CS/BMP-2@SF)

BMP-2 SF solution at a final concentration of 2% (w/v) was prepared by dissolving BMP-2 in SF solution. Then, TGF-β1@CS NPs were added. The TGF-β1@CS/BMP-2@SF of different concentrations was constructed by ultrasound-induced crosslinking. TGF-β1@CS/BMP-2@SF (5%, 10%, 15%, and 20%) was synthesized by volume ratio of TGF-β1@CS and BMP-2@SF at 1:20, 1:10, 3:20, and 1:5, which was then stored in a − 4 °C freezer.

A SEM was applied for morphology observation. The mechanical property was assessed by a universal mechanical tester. The release of TGF-β1 and BMP-2 in vitro was evaluated by ELISA. The in vitro degradation and water absorption of composite system were analyzed by weighing.

### Biocompatibility examination of TGF-β1@CS/BMP-2@SF

The complete medium was prepared by the addition of 10% fetal bovine serum and 1% penicillin-streptomycin into high-glucose Dulbecco’s modified Eagle’s medium. BMSCs were then seeded in 10-cm petri dish supplemented with complete medium and cultured in an incubator at 37 °C with 5% CO_2_. The medium was renewed every 3 days.

Five sets of TGF-β1@CS/BMP-2@SF and CS/SF of equal sizes were collected, immersed in complete medium for 3 days, and filtered through a 0.22-μm sterile needle filter. The extracts of TGF-β1@CS/BMP-2@SF and CS/SF were then obtained. BMSCs were seeded in a 96-well plate at a density of 2000 cells/well and cultured in a 5% CO_2_ incubator at 37 °C under conditions of complete medium (the control group), CS/SF extract (the CS/SF group), and TGF-β1@CS/BMP-2@SF extract (the TGF-β1@CS/BMP-2@SF group). Cell counting kit-8 (CCK-8) assay was performed on the 1st, 3rd, 5th, and 7th day respectively to assess cell viability. Briefly, the medium in the 96-well plate was removed 2 h before detection. Following washing with phosphate buffer saline (PBS), every 10 μL CCK-8 reagent and 100 μL complete medium were added into each well, followed by 2-h incubation in a 96-well plate. The supernatant was then collected and placed in a new 96-well plate. The absorbance value at a wavelength of 450 nm was measured using a multi-wavelength microplate reader.

The extracts of TGF-β1@CS/BMP-2@SF and CS/SF were obtained and used for culture of BMSCs in a 96-well plate at a density of 2000 cells/well. BMSCs cultured in the complete medium served as the control group. The medium was removed on the 1st, 3rd, 5th, and 7th day. After 3 PBS washes, the cells (200 μL) were then subjected to live/dead cell double staining in 10 mL PBS containing 5 μL Calcein and 20 μL ethidium homodimer. Following incubation at room temperature for 30 min, the dye liquor was removed. Therefore, the cells were rinsed 3 times by PBS and photographed.

BMSCs were seeded on the surface of TGF-β1@CS/BMP-2@SF and CS/SF while CS/SF without inoculation of BMSCs served as the control group. The medium was removed on the 1st, 3rd, 5th, and 7th day. After PBS washing, the cells were fixed in 4% paraformaldehyde for 30 min, dehydrated and subjected to critical point drying. Metal spraying was performed using ion sputter coater. The adhesion of BMSCs on the surface of TGF-β1@CS/BMP-2@SF and CS/SF was observed under the SEM with images acquired. Cell diffusion area was analyzed using the Image Pro Plus 7.0 software, with at least 30 cells in each sample analyzed.

### In vitro chondrogenesis evaluation of TGF-β1@CS/BMP-2@SF

TGF-β1@CS/BMP-2@SF and CS/SF were respectively co-cultured with BMSCs. The medium was renewed every 3 days. Histological staining of Safranin O was performed on the 28th day. Briefly, 2.5 g Safranin O was dissolved in 100 mL of 95% ethanol to prepare 2.5% Safranin O ethanol solution as storage solution, and then diluted to 0.5% Safranin O aqueous solution when used. The cells were then subjected to 0.5% safranin O staining for 1 min, air-dried, permeabilized with xylene, and blocked with neutral gum. If the staining time was too short, the staining was not enough; if the time was more than 1 min, the staining effect was equal to 1 min of staining. Meanwhile, the immunohistochemical staining of collagen II was performed on the 28th day. In short, the cells were subjected to antigen retrieval by trypsin, blocked with 5% serum, and then incubated overnight with mouse anti-human type II collagen antibody (1:500, Sigma-Aldrich Chemical Company, St Louis, MO, USA). Immunohistochemical staining was performed according to the instructions of immunohistochemistry kit and 3,3′-diaminobenzidine (DAB) chromogenic kit. The positive area was observed brown under a microscope. The culture solution was collected on the 7th, 14th, 21st, and 28th days to measure the content of glycosaminoglycan (GAG) by Alcian blue colorimetric method at a wavelength of 490 nm. Chondroitin sulfate served as a reference standard.

TGF-β1@CS/BMP-2@SF and CS/SF were respectively co-cultured with BMSCs for 14 days. The cells were then collected to determine the expression of Aggrecan and collagen II with β-actin serving as the loading control. Total RNA was extracted from cells according to the manual of TRIzol reagents. The absorbance value of RNA in each group was measured. With 5 μL total RNA as template, fragments were amplified using reverse transcription quantitative polymerase chain reaction (RT-qPCR) kits. The primer sequences are shown in Table 1. The reaction conditions for β-actin were at 94 °C for 3 min, at 94 °C for 30 s, at 59 °C for 30 s, at 72 °C for 30 s, and at 72 °C for 10 min, 30 cycles in total. The reaction conditions for Aggrecan were at 94 °C for 3 min, at 94 °C for 30 s, at 60 °C for 30 s, at 72 °C for 30 s, and at 72 °C for 10 min, 30 cycles in total. The reaction conditions for collagen II were at 94 °C for 3 min, at 94 °C for 30 s, at 55 °C for 30 s, at 72 °C for 30 s, and at 72 °C for 10 min, 30 cycles in total. Then, 3 μL amplified products were collected and subjected to 2% agarose gel electrophoresis. The average gray values were determined by the Gel-Pro Analyzer 4 software. The relative mRNA expression equaled to the ratio of the gray value of genes to be tested to that of β-actin.

**Table 1:** The primer sequences used for RT-qPCR

RNA	Primer sequence (5′-3′)	Length
β-actin	Forward: 5′-ATCGTGCGTGACATTAAGG-3′	478
	Reverse: 5′-GGCCGGACTCGTCATACTCC-3′	
Aggrecan	Forward: 5′-CACTGTTACCGCCACTTCCC-3′	300
	Reverse: 5′-GACATCGTTCCACTCGCCCT-3′	
Collagen II	Forward: 5′-ACACTCAAGTCCCTCAACA-3′	131
	Reverse: 5′-TCAATCCAGTAGTCTCCACTCT-3′	

### In vivo chondrogenesis evaluation of TGF-β1@CS/BMP-2@SF

In total, 18 qualified New Zealand white rabbits (aged 6 weeks, weighing 1.52–2.23 kg, Laboratory Animal Center, Academy of Military Medical Sciences, Beijing, China) of either sex were enrolled in our study and anesthetized. A vertical incision was made on the inside of both knee joints to expose the articular surface by cutting open the skin, subcutaneous tissues, deep fascia, and articular capsule. The full-thickness defects of articular cartilage (4 mm in diameter, 3 mm in depth) through subchondral bone plate were created by drilling a hole on the articular surface of weight-bearing area in the medial femoral condyle [15]. The 36 keen joints of 18 rabbits were randomly divided into 6 groups, the blank group (keen joints treated with normal saline), the CS/SF group (keen joints treated with CS/SF), the TGF-β1 group (keen joints treated with TGF-β1), the BMP-2 group (keen joints treated with BMP-2), the TGF-β1/BMP-2 group (keen joints treated with TGF-β1/BMP-2), and the TGF-β1@CS/BMP-2@SF group (keen joints treated with TGF-β1@CS/BMP-2@SF). The incision was sutured layer by layer. On the 3rd day post operation, penicillin (80 TU) was administered through intramuscular injection. Rabbits were separately caged with freedom of action and euthanized by anesthesia at the 6th and 12th weeks after operation. The knee joints were collected and observed.

### Pathological and histomorphological observation by hematoxylin-eosin (HE) staining

The knee joints were evaluated and quantified using the Wakitani score [16] under a dissecting microscope. According to the International Cartilage Repair Society (ICRS) [17], knee joints were scored regarding the morphology, surface regularity and thickness of repair tissues, integration of donor with adjacent host cartilage, and collagen II staining within repair tissues (Table 2). The maximum score was 14 points and lower score indicated that repair tissues were closer to normal cartilage.

**Table 2:** Histological grading scale for the defects of cartilage

Category	Score
Cell morphology	
Hyaline cartilage	0
Mostly hyaline cartilage	1
Mostly fibrocartilage	2
Mostly non-cartilage	3
Non-cartilage only	4
Matrix staining	
Normal	0
Slightly reduced	1
Markedly reduced	2
No metachromatic stain	3
Surface regularity*	
Smooth (> 3/4)	0
Moderate (> 1/2–3/4)	1
Irregular (1/4–1/2)	2
Severely irregular (< 1/4)	3
Thickness of cartilage^#^	
> 2/3	0
1/3–2/3	1
< 1/3	2
Integration of donor with adjacent host cartilage	
Both edges integrated	0
One edge integrated	1
Neither edge integrated	2
Total maximum	14

*Total smooth area of the reparative cartilage compared with the entire area of the cartilage defect

^#^Average thickness of the reparative cartilage compared with that of the surrounding cartilage

HE staining was performed as follows. A part of knee joint tissues from each group were fixed with 4% formaldehyde for 6 h, paraffin-embedded and sliced into 5-μm serial sections. The sections were then baked at 60 °C overnight, dewaxed with xylene I for 20 min and xylene II for 20 min, and dehydrated with ethanol of descending concentrations, followed by hematoxylin staining for 10 min. After washing under running water for 15 min, the sections were blued by water washing. The sections were subsequently counterstained with eosin for 30 s, dehydrated by ethanol, permeabilized by xylene, and mounted with neutral gum. Finally, the sections were observed under an optical microscope (NIKON, Tokyo, Japan) and photographed.

### Statistical analysis

SPSS 21.0 software (IBM Corp., Armonk, NY, USA) was used for data analysis. Measurement data were presented as mean ± standard deviation. Data between two groups of unpaired design were compared by unpaired *t* test, while data among multiple groups were analyzed by one-way analysis of variance (ANOVA), followed by Tukey’s test. Data at various time points were analyzed by Bonferroni-corrected repeated measures ANOVA. A value of *p* < 0.05 was considered to be indicative of statistical significance.

## Results

### Successful construction of TGF-β1@CS

**Fig. 1:**
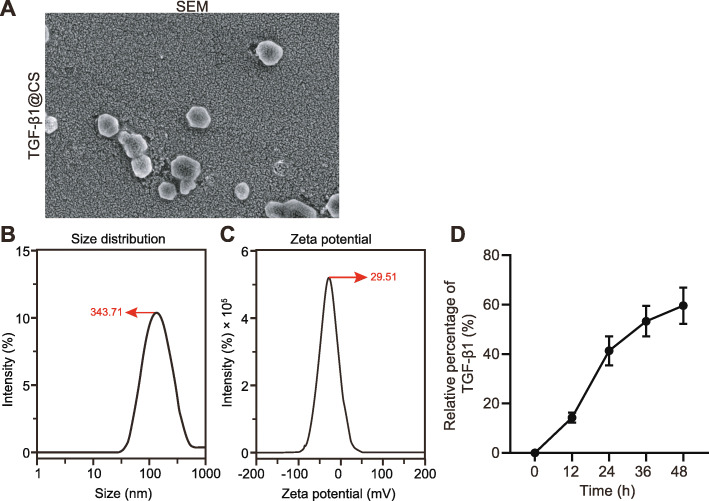
Characterization of TGF-β1@CS NPs. **a** SEM images of TGF-β1@CS NPs. **b** Dynamic light scattering analysis for size distribution of TGF-β1@CS NPs. **c** Zeta potential of TGF-β1@CS NPs. **d** In vitro release of TGF-β1 measured by ELISA. The results were measurement data and expressed as mean ± standard deviation derived from at least 3 independent experiments. Data comparison among multiple groups was analyzed by one-way ANOVA, followed by Tukey’s post hoc test

The size distribution, zeta potential, morphology, and internal structure of TGF-β1@CS and release of TGF-β1 in vitro were detected to identify the construction of TGF-β1@CS. TGF-β1@CS was observed with round appearance (Fig. 1a). As revealed from dynamic light scattering (Fig. 1b), size distribution of TGF-β1@CS presented with a single peak, the mean size distribution was 343.71 ± 20.48 nm, and polydispersity index (PDI) was 0.224 ± 0.142. Due to the presence of positive charge, the mean zeta potential of TGF-β1@CS was 29.51 ± 3.24 mV (Fig. 1c). In addition, TGF-β1 released from TGF-β1@CS lasted for approximately 48 h in vitro (Fig. 1d). Taken together, these results indicated the successful construction of TGF-β1@CS.

### Successful construction of BMP-2@SF

**Fig. 2:**
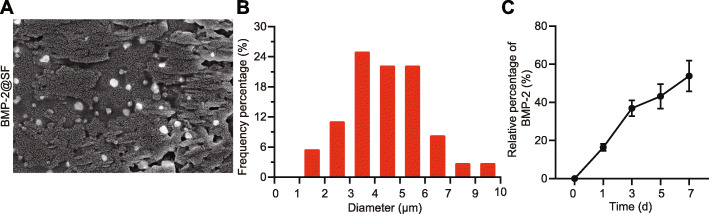
Characterization of BMP-2@SF hydrogel. **a** SEM images of BMP-2@SF hydrogel (× 400). **b** Pore diameter of BMP-2@SF hydrogel. **c** In vitro release of BMP-2 measured by ELISA. The results were measurement data and expressed as mean ± standard deviation derived from at least 3 independent experiments. Data comparison among multiple groups was analyzed by one-way ANOVA, followed by Tukey’s post hoc test

According to microscopic observation of morphology (Fig. 2a), uniform micron holes were detected in BMP-2@SF with thin walls in relatively neat structure. Through further analysis using the Image J software (Fig. 2b), the mean diameter of hydrogel holes was found to be 5.34 ± 1.52 μm. In addition, BMP-2 was found to be continuously released for approximately 7 days in vitro (Fig. 2c). These results demonstrated the successful construction of BMP-2@SF.

### Characterization of TGF-β1@CS/BMP-2@SF

**Fig. 3:**
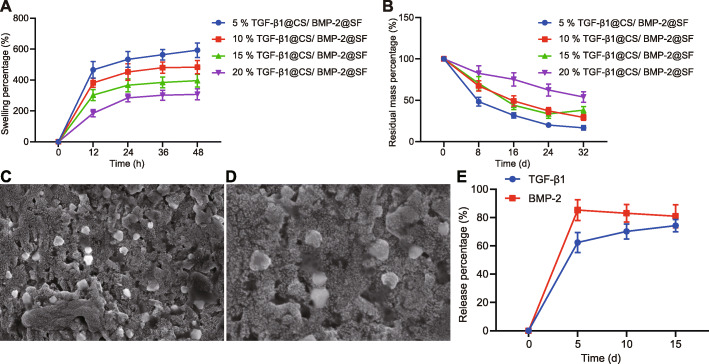
Characterization of TGF-β1@CS/BMP-2@SF and 15% TGF-β1@CS/BMP-2@SF. **a** Water absorption of TGF-β1@CS/BMP-2@SF of different concentrations. **b** In vitro degradation of TGF-β1@CS/BMP-2@SF of different concentrations. **c** Water absorption of 15% TGF-β1@CS/BMP-2@SF. **d** In vitro degradation of 15% TGF-β1@CS/BMP-2@SF. **e** In vitro release of TGF-β1 and BMP-2 from 15% TGF-β1@CS/BMP-2@SF. The results were measurement data and expressed as mean ± standard deviation derived from at least 3 independent experiments. Data comparison at different time points was analyzed by Bonferroni-corrected repeated measures ANOVA

TGF-β1@CS/BMP-2@SF of different concentrations was constructed, followed by assessment of in vitro degradation and water absorption. The concentration of BMP-2 remained constant. As depicted in Fig. 3a, b, both in vitro degradation and water absorption of composite system decreased over the increase of hydrogel concentration. The sequence of water absorption was 20% <  15% <  10% < 5%, while the sequence of degradation was 5% >  15% >  10% >  20%. Considering the body fluid exchange ability between implants and external environment and integrity-holding property of composite system, 20% TGF-β1@CS/BMP-2@SF was selected as the ideal composite system for subsequent experiments.

Further observation under the SEM verified that TGF-β1@CS NPs were successfully incorporated in 15% TGF-β1@CS/BMP-2@SF (Fig. 3c, d).

Subsequently, in vitro release of TGF-β1 and BMP-2 from hydrogel was detected by ELISA (Fig. 3e). The 15% TGF-β1@CS/BMP-2@SF was constructed by introducing TGF-β1@CS NPs into BMP-2@SF hydrogel, leading to promoted release property in vitro. When TGF-β1 release time in vitro was extended from 48 h to approximately 10 days, no significant effects were observed regarding the in vitro release of BMP-2. The above findings identified that TGF-β1@CS/BMP-2@SF was successfully constructed in our study.

### TGF-β1@CS/BMP-2@SF exhibited favorable biocompatibility

**Fig. 4:**
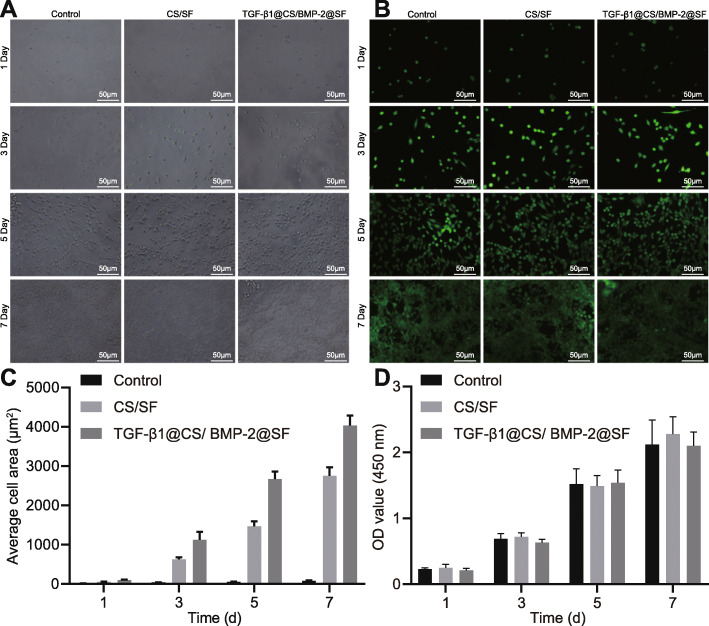
Biocompatibility characterization and assessment of 15% TGF-β1@CS/BMP-2@SF. **a** Cell adhesion of 15% TGF-β1@CS/BMP-2@SF (× 200). **b** Live/dead cell double staining of 15% TGF-β1@CS/BMP-2@SF (× 200). **c** Cell adhesion in the presence of TGF-β1@CS/BMP-2@SF. **d** Cell viability of BMSCs detected by CCK-8 assay in the presence of TGF-β1@CS/BMP-2@SF. The results were measurement data and expressed as mean ± standard deviation derived from at least 3 independent experiments. Data comparison at different time points was analyzed by Bonferroni-corrected repeated measures ANOVA. *n* = 12

As illustrated in Fig. 4a, BMSCs exhibited normal morphology on the surface of hydrogel composite system accompanied by obvious cell proliferation. The results of live/dead cell double staining (Fig. 4b) showed that extracts of hydrogel composite system did not significantly affect proliferation of BMSCs. Compared with the control group, cell adhesion was increased significantly in the CS/SF and TGF-β1@CS/BMP-2@SF groups (Fig. 4c). Moreover, quantification analysis regarding cell viability by CCK-8 assay revealed no significant differences among 3 groups (Fig. 4d), corresponding with the results of SEM photograph and live/dead cell double staining, suggesting the favorable biocompatibility.

### TGF-β1@CS/BMP-2@SF exhibited chondrogenic ability

**Fig. 5:**
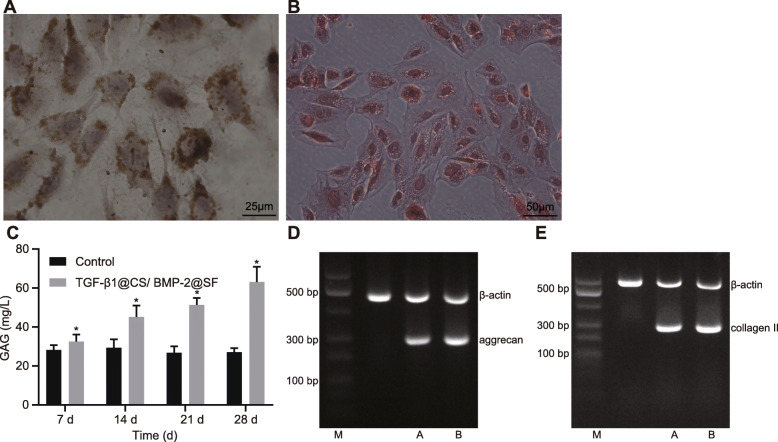
**a** Immunohistochemical staining of collagen II at the 4th week of co-culture of TGF-β1@CS/BMP-2@SF with BMSCs (× 400). **b** Safranin O staining at the 4th week of co-culture of TGF-β1@CS/BMP-2@SF with BMSCs (× 200). **c** The GAG content increases in the presence of 15% TGF-β1@CS/BMP-2@SF detected by Alcian blue colorimetric method. **d** The expression of Aggrecan and collagen II in the CS/SF group normalized to β-actin determined by agarose gel electrophoresis. **e** The expression of Aggrecan and collagen II in the TGF-β1@CS/BMP-2@SF group normalized to β-actin determined by agarose gel electrophoresis. The results were measurement data and expressed as mean ± standard deviation derived from at least 3 independent experiments. Data comparison between two groups was analyzed by unpaired *t* test. *n* = 12

The immunohistochemical staining of collagen II showed obvious cartilage lacuna in cell mass formed by co-culture with TGF-β1@CS/BMP-2@SF along with nucleus and surrounding purple-stained extracellular matrix accumulation (Fig. 5a). Furthermore, cartilage specific matrix staining was observed by Safranin O staining, suggesting the rich accumulation of proteoglycan in the extracellular matrix (Fig. 5b). The subsequent quantitative analysis of GAG content demonstrated that difference among groups became more significant over the time of co-culture (Fig. 5c).

Agarose gel electrophoresis was followed to verify the expression of genes to be test in vitro (Fig. 5d, e), indicating the chondrogenic ability of hydrogel composite system in vitro.

### TGF-β1@CS/BMP-2@SF reduced histological scores

At the 6th and 12th weeks after operation, histological score was significantly lower in the other 2 groups than that in the control group at the same time (*p* < 0.05), while the TGF-β1@CS/BMP-2@SF group had much lower score than that in the CS/SF group (*p* < 0.05). Additionally, significantly reduced histological scores were observed in each group at the 12th week over time in comparison to the 6th week after operation (*p* < 0.05) (Table 3). These findings indicated that TGF-β1@CS/BMP-2@SF could alleviate histology.

**Table 3:** Comparison of histological scores at the 6th and 12th weeks after operation (*n* = 12)

Time point	Control	CS/SF	TGFβ1	BMP2	TGFβ1/BMP2	TGF-β1@CS/BMP-2@SF
The 6th week	11.00 ± 1.28	8.58 ± 1.08*	7.25 ± 1.96*	8.08 ± 1.31*	5.58 ± 1.24*&	5.67 ± 0.78*^#^
The 12th week	9.92 ± 1.08	5.92 ± 0.79*	4.75 ± 1.22*	5.42 ± 1.98*	3.17 ± 1.11*&	2.33 ± 0.49*^#^

The results were measurement data and expressed as mean ± standard deviation. Data comparison among multiple groups was analyzed by one-way ANOVA, followed by Tukey’s post hoc test. The experiment was repeated 3 times independently

*p < 0.05 vs. the control group at the same time

^#^*p* < 0.05 vs. the CS/SF group at the same time

### TGF-β1@CS/BMP-2@SF improved pathological changes

Postoperative morphological views at the 6th week (Fig. S1) showed sunken and unsmooth cartilage defect areas in the control group with poor integrity between repair areas and adjacent cartilage as well as more inflammatory cell infiltration. The repair effect of the CS/SF, TGF-β1, BMP-2, and TGF-β1/BMP-2 groups was better than the control group. The cartilage defect areas were mostly well filled, most repair tissues were similar to fibrous cartilage, the surface of repair tissues was basically smooth, the integrity between repair tissues and adjacent tissues was better with obvious fracture, and inflammatory cell infiltration decreased. The TGF-β1@CS/BMP-2@SF group exhibited the best repair effect among the 3 groups. The cartilage defect areas were filled with hyaline cartilage, BMSCs differentiated to chondrocytes, the surface of most repair tissues was smooth accompanied by fracture with adjacent tissues, and inflammatory cell infiltration further decreased.

After another 6 weeks (Fig. S1), the repair effect was worst in the control group where defect areas were mainly filled with fibrous tissues, the surface of most repair tissues was rough and obvious fracture occurred between repair tissues and adjacent cartilage. The number of infiltrated inflammatory cells was larger compared with the 6th week. In the CS/SF, TGF-β1, BMP-2, and TGF-β1/BMP-2 groups, defect areas were mostly filled with hyaline cartilage and repair tissues with basically smooth surface were well integrated with adjacent cartilage. The number of infiltrated inflammatory cells was smaller compared with the 6th week. The superior repair effect was still observed in the TGF-β1@CS/BMP-2@SF group since defect areas were completely filled with hyaline cartilage and repair tissues with smooth surface were completely integrated with adjacent cartilage. Only a small number of inflammatory cells were infiltrated. To conclude, TGF-β1@CS/BMP-2@SF could exert superior repair effect on articular cartilage defects.

## Discussion

**Fig. 6:**
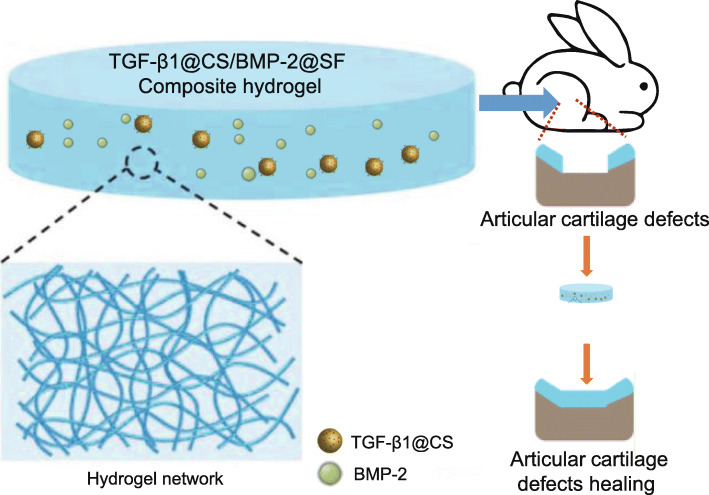
The potential of TGF-β1@CS/BMP-2@SF to repair articular cartilage defects through controlling in vitro release of TGF-β1 and BMP-2

Articular cartilage, only populated by chondrocytes, is usually difficult to repair or regenerate while tissue engineering by means of biomaterials has been attracting great attention with regard to its therapeutic significance [18]. Accumulating evidence has shown that SF and CS possess favorable bioactivity and physical properties implication in tissue engineering for human [19, 20]. During the current investigation, attempts were made to reveal the functional role of TGF-β1@CS/BMP-2@SF in repairing articular cartilage defects of the knee joints. Collectively, the experimental data demonstrated that TGF-β1@CS/BMP-2@SF conferred protection against articular cartilage defects in the knee joints by enhancing the chondrogenic capability of BMSCs both in vitro and in vivo (Fig. 6).

At the initial stage of our experiment, TGF-β1 or BMP-2 was successfully loaded in CS NPs and SF hydrogel, respectively. Tissue regeneration can be described as a wound healing process depending on mediation of various growth factors, primarily TGF-β1, which has been reported to be functional for cutaneous wound healing through curcumin NPs incorporated with collagen-CS scaffold [21]. Hydrogels have been identified as an outstanding option for cartilage regeneration through introduction of chondrocytes or their precursors in a highly swollen polymer network, allowing shuttle of nutrients and biological molecules [22]. The modular CS hydrogels in combination with TGF-β1 have been elucidated in contributing to clinical efficacy and safety of chondroinductive therapies [8]. CS-coated SF electrospun scaffolds loaded with BMP-2 polypeptide-functionalized graphene oxide have been proposed to promote bone defect regeneration [23]. More importantly, TGF-β1 or BMP-2 has been indicated to be incorporated in glycol CS hydrogel to promote bone formation in a rat model of tibial defect [9]. Hence, subsequent efforts were made to incorporate TGF-β1@CS and BMP-2@SF together for investigation purpose.

Further experimental data regarding successfully incorporated TGF-β1@CS and BMP-2@SF showed that TGF-β1@CS/BMP-2@SF was featured with favorable biocompatibility and superior bone formation abilities at the defect site as evidenced by potentiated chondrogenic ability of BMSCs. CS/SF sponge has been highlighted to hold promises for cartilage tissue engineering characterized by remarkable biocompatibility and chemistry versatility to facilitate chondrocyte-like cell adhesion, proliferation, and matrix production [24]. Biocompatibility is of great importance when fabricating cellular scaffolds based on hydrogel for bioengineered tissue generation [25]. In addition, hydrogels have been validated to give rise to chondrogenic differentiation of BMSCs during cartilage tissue engineering [26]. Similar to our study, a previous study reported that cartilage defects in the knee joints of rabbits can be improved by SF/CS scaffold combined with BMSCs [27]. Furthermore, BMSCs expressing TGF-β1 have been observed to potentiate cartilage defect repair and the impaired cartilage is composed of a larger number of GAG and collagen II [28]. Consistently, the content of GAG and expression levels of collagen II and Aggrecan were elevated in BMSCs co-cultured with TGF-β1@CS/BMP-2@SF in our study, indicating improved cartilage defects induced by TGF-β1@CS/BMP-2@SF. Largely in agreement with our findings, TGF-β1 has the capacity to upregulate the expression of collagen II and Aggrecan in chondrocytes of rats [29]. Also, in the presence of BMP-2 under conditions of collagen sponge scaffolds, chondrocytes are found to be re-differentiated and metabolically active in parallel with collagen IIB and Aggrecan molecules as well as the synthesis of hyaline-like cartilage matrix [30]. As reported in a previous study, BMP-2 at a dose of 5 μg results in cartilage of higher quality with promoted surface regularity, tissue integration, and higher levels of collagen II and Aggrecan at the 2nd week after scaffold implantation [31], suggesting the need of further studies to identify the optimal doses of TGF-β1 and BMP-2 for better clinical outcomes.

To conclude, the present study focused on the superior repair effects of TGF-β1@CS/BMP-2@SF as an emerging remedy on articular cartilage defects of the knee joints. TGF-β1@CS/BMP-2@SF was demonstrated to show remarkable biocompatibility and potentiated chondrogenesis of BMSCs as revealed from a cell model as well as a rabbit model. Herein, we reported that TGF-β1@CS/BMP-2@SF may have the potential of clinical use for the articular cartilage defects, conducive to generating more and better targeted therapies. However, attention should be paid to the physiological and patho-physiological differences when relating the animal results to the human clinical setting. Further experiments on the diagnosed human are required to determine the value of this new clinical application in detecting articular cartilage defects.

## Supplementary Information

**Additional file 1: Fig. S1.** HE staining of cartilage tissues at the 6th and 12th weeks after operation (× 400).

## Data Availability

The datasets generated and/or analyzed during the current study are available from the corresponding author on reasonable request.
